# Natural history of *Echinococcus granulosus* microcyst development in long term *in vitro* culture and molecular and morphological changes induced by insulin and BMP-4

**DOI:** 10.3389/fvets.2022.1068602

**Published:** 2023-01-09

**Authors:** Ali Derakhshani, Seyed Mohammad Mousavi, Masoud Rezaei, Ali Afgar, Ali Reza Keyhani, Mohammad Ali Mohammadi, Shahriar Dabiri, Majid Fasihi Harandi

**Affiliations:** ^1^Research Center for Hydatid Disease in Iran, Kerman University of Medical Sciences, Kerman, Iran; ^2^Student Research Committee, Kerman University of Medical Sciences, Kerman, Iran; ^3^Leishmaniasis Research Center, Kerman University of Medical Sciences, Kerman, Iran; ^4^Department of Pathology, Afzalipour Medical School, Pathology and Stem Cells Research Center, Kerman University of Medical Sciences, Kerman, Iran

**Keywords:** degeneration, calcification, apoptosis, growth factor, bone morphogenetic protein (BMP), hydatid disease

## Abstract

**Introduction:**

Cystic echinococcosis (CE) caused by the cestode *Echinococcus granulosus* is a disease of worldwide public health and economic importance. The determinants and underlying cellular mechanisms of CE development and fate in intermediate hosts are largely unknown. Hormones and cytokines such as insulin and BMP-4 are the key players in the development, differentiation, and apoptosis. In this study, we evaluated the long term natural history of *E. granulosus* microcysts in an vitro setting and the molecular and morphological changes induced by the growth factors, insulin and BMP4 during the development of metacestode stage of *E. granulosus*.

**Methods:**

*E. granulosus* protoscoleces were cultivated and the parasite development was followed in the long term mono-phasic culture for 105 days and the morphometric, molecular and immunohistochemical changes were evaluated, including the microcysts number and size, microcysts development and deformation rates as well as the markers of calcification (Alizarin Red staining) and apoptosis (BAX, BCL2, Caspase-3, Caspase-8 and TNF-α expression) in the microcysts. Also the biological, histological and molecular consequences of insulin and BMP-4 treatment on the parasite development were evaluated.

**Results:**

Insulin and BMP-4 treatment of microcysts resulted in significant increase in microcyst formation, increased size, reduced apoptosis and deformation of the microcysts. Alizarin red staining of the microcysts treated with the insulin and BMP-4 confirmed that calcium deposition is significantly lower than the untreated microcysts. Also Alizarin Red staining and Immunohistochemistry of the microcysts indicates that calcium accumulation in deformed microcysts is higher than the normal ones on day 105. The microcysts began to wrinkle and the germinal layer was partially detached from the laminated layer on day 84.

**Conclusion:**

Results of the present study suggest that the degenerative changes in hydatid cysts can be slowed down by insulin and BMP-4, indicating that cellular factors and host hormones could contribute to the longevity of hydatid cysts. Significant evidences are provided suggesting that the microcysts cultivated *in vitro* can undergo calcification and apoptotic processes similar to what have been observed in the natural hydatid infection in the intermediate hosts.

## 1. Introduction

Cystic echinococcosis (CE) is a zoonotic disease caused by the larvae of the canine tapeworm *Echinococcus granulosus*, which is a disease of widespread public health and economic importance. The life cycle of *E. granulosus* is complex involving two mammalian hosts, a carnivore definitive host and a herbivore intermediate host. Human is accidental dead-end host harboring the metacestode stage of the parasite known as hydatid cyst in the liver and lungs. This parasite has a worldwide distribution and it has been estimated to infect 2–3 million people across the globe (1, 2).

Infection of human and intermediate hosts occurred through ingestion of infective eggs, which are released in the feces of the definitive hosts. The oncospheres released in the gastrointestinal tract, penetrate the intestinal wall and migrate *via* the portal system to various internal organs and tissues, mainly liver and lungs, where they develop into hydatid cyst. Hydatid cyst is a unilocular fluid-filled cyst comprised of an inner nucleated germinal layer (GL) responsible for production of the infective stage, a structure called protoscolex (PSC), an outer acellular laminated layer (LL) that is unique to the genus *Echinococcus*, and an external host-produced adventitial layer as the result of immune response to the parasite. Upon feeding on infected organs, sexual maturity of adult *E. granulosus* occurs in the small intestine of the carnivorous definitive host within 4–5 weeks. The eggs and/or gravid proglottids are disseminated in the environment *via* dog defecation (3).

According to the current knowledge, cyst development in human can be divided into four stages: (1) the maturing phase (cyst and protoscoleces development) (2) stable phase (cyst growth and exponential increase in cyst volume); (3) unstable phase (germinal layer detachment and damage to parasite tissues); (4) degenerative phase (cyst calcification and death). Understanding the molecular and biological determinants of hydatid cyst development is crucial in the diagnosis and management of CE. Unfortunately our knowledge on the events during developmental process of hydatid cysts is poor (4).

The bi-directional nature of *E. granulosus in vitro* cultivation in which the parasite develops either into the strobilated worm in di-phasic culture media or to the microcysts in mono-phasic media, is a fascinating feature and a valuable tool for understanding *Echinococcus* biology. Understanding the molecular basis and determinants of the parasite development improves our knowledge on the natural history of human CE (5–7).

Very little is known about the natural history of cyst development in long term *in vitro* culture as well as the molecular mechanisms involving the development and survival of the parasite. Of special interest is the extensive studies of J.D.Smyth in 1960–70's and his significant contribution in our knowledge of *E. granulosus*, leading to the identification of host biochemical and physiological factors. Nonetheless, limited studies have been performed to identify these factors at molecular level. It is believed that several host-derived factors including different growth factors, hormones, cytokines as well as host-parasite cell interactions influence the natural history of the cyst (8).

Most signaling systems are evolutionarily conserved in most animal cell-cell communications. These systems include hormones and cytokines such as insulin, epidermal growth factor (EGF), fibroblast growth factor (FGF), membrane-bound receptor tyrosine kinases (RTKs) and growth factor-converting cytokines (TGF-b) / Bone morphogenetic protein (BMP) (9–11).

Insulin signaling has been studied commonly in mammals. Invertebrates such as *Caenorhabditis elegans* and *Drosophila melanogaster* have been used as model organisms for studying insulin signaling because of its crucial role in regulating metabolic processes, growth control, reproduction, and aging (12–14). Insulin acts by binding to the receptors located at the surface of target cells. Tyrosine kinases have already been identified in a wide variety of parasitic helminth organisms, including *E. multilocularis* (15, 16), *Schistosoma mansoni* (17, 18) and *S. japonicum* (19). Hemer et al. studied the *in vitro* effects of human insulin on *E. multilocularis* metacestodes, and suggested that the host insulin stimulates the growth and development of the metacestodes (16). It has been shown that peptide-like insulins or host insulins can stimulate insulin receptors in the helminths through the main signaling pathways of extracellular signal-regulated kinase (Erk)/mitogen-activated protein kinases (MAPK) as well as the serine/threonine kinase Akt (also known as protein kinase B)/phosphoinositide-3-kinase (PI3K) sub-pathways. Moreover, due to the high concentration of insulin in the liver, insulin can probably play a significant role in the establishment of oncospheres and the growth and development of *E. granulosus*, as it was shown in other closely related platyhelminth species including *E. multilocularis* and *S. japonicum* (8, 16, 20).

A multifunctional growth factor, bone morphogenetic proteins (BMPs) belong to the transforming growth factor b (TGFb) superfamily. Recent studies have comprehensively examined BMPs role in embryonic development and cellular function. Smad1, 5, and 8 are the downstream molecules of BMP receptors and play a key role in BMP signal transduction (21). BMPs in metazoan have an important role in the development, differentiation, apoptosis in helminths and many studies have focused on the role of BMP subfamily on *S. mansoni* development (22) and *E. multilocularis* (23, 24). Findings of a study on metacestodes of *E. multilocularis* Smad E, indicated that it is expressed in all larval stages of the parasite and is involved in TGF-b and BMP signaling (23). Studies have shown that one or more parasite TGF-b/BMP receptors can functionally interact with host TGF. Therefore, biochemical studies on the interaction between *Echinococcus* TGF-b/BMP receptors and host TGF-b should be given more attention (8). However, our knowledge on the effect of BMP on *E. granulosus* growth and development is limited and very little is known about the role of this growth factor on the parasite.

The aim of this study was to evaluate the natural history of *E. granulosus* in long term *in vitro* culture and the effect of insulin and BMP4 growth factors on the development and growth of this parasite.

## 2. Materials and methods

**Figure 1:**
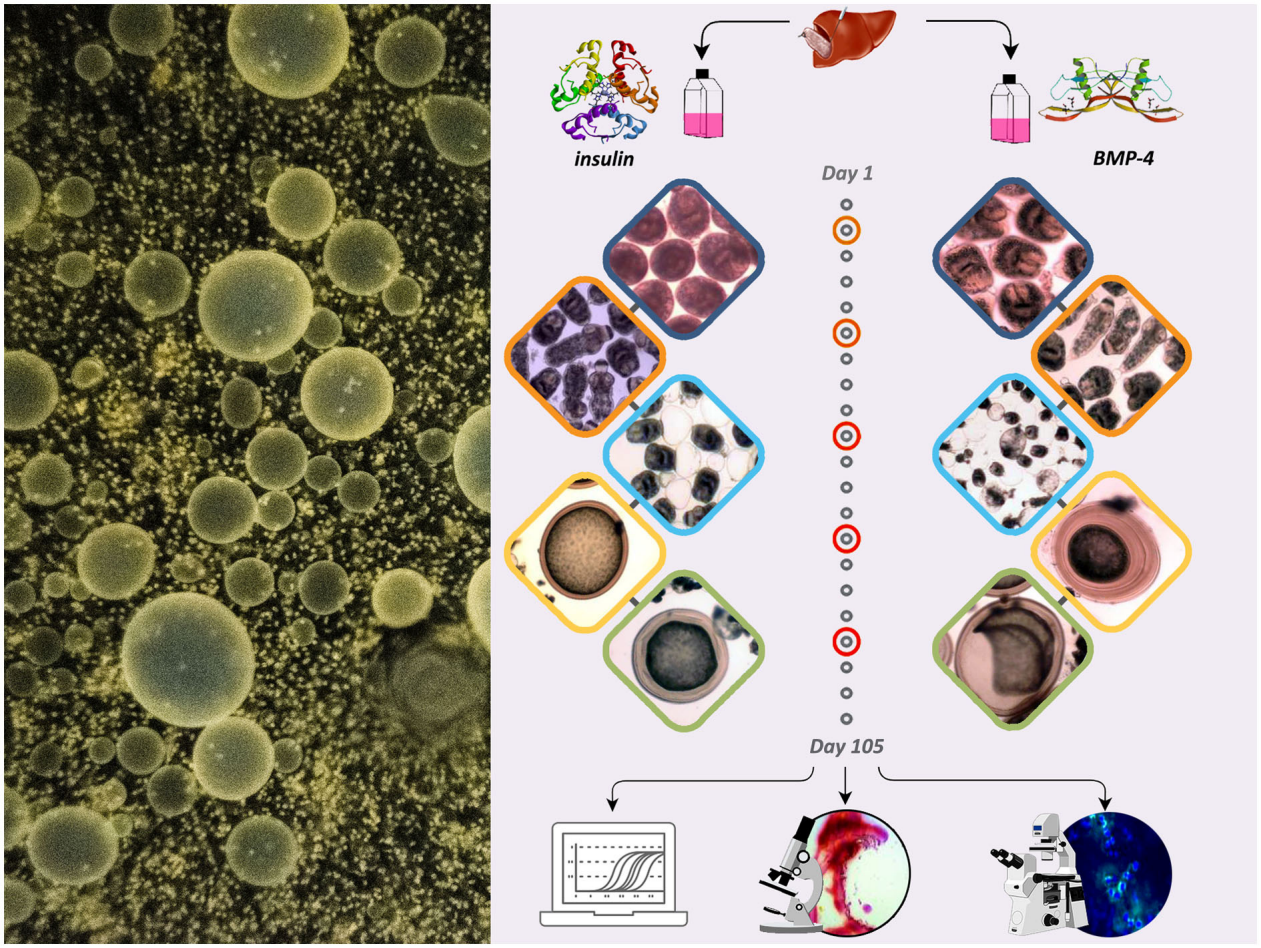
The schematic presentation of the study showing the effect of insulin and BMP-4 on the growth and development of *E. granulosus* cultured *in vitro* for 105 days.

The schematic presentation of the study with the following stages is demonstrated in Figure 1.

### 2.1. Parasite culture and preparations

*Echinococcus granulosus* cysts were obtained by dissection of livers from naturally infected sheep slaughtered under the supervision of the veterinary officers in Kerman municipal abattoir. The infected organs were immediately transferred to the parasitology laboratory and under sterile conditions the hydatid fluid containing protoscoleces was aspirated with a 50 ml syringe and the laminated/germinal layer were removed. The protoscoleces were carefully washed five times with phosphate-buffered saline containing 100 U/ml penicillin and 100 μg/ml streptomycin (PBS-PS). Before cultivation, the viability of the protoscoleces was checked by 0.1% eosin test under a light microscope (25). The protoscoleces were genotyping of was performed using mitochondrial cox1 PCR sequencing and the sequence was submitted to GenBank.

Aliquots of 2000 protoscoleces with a minimal viability of 95% were used for *in vitro* culture in monophasic media to reach the microcyst forms, according to the method described elsewhere (26). Briefly, the protoscoleces were separated from brood capsules by treating in 0.1% (w/v) pepsin produced in 0.85% (w/v) sodium chloride, pH 4.0 for 30 min at 37°C. The protoscoleces were subsequently added into monophasic DMEM and the media were changed every 4 days and viability and morphological development of the parasite was monitored under an invert microscope (TCM 400, Labomed Inc., CA).

BMP-4 (Sigma-Aldrich, USA) stock solutions was prepared in PBS with a concentrations 100 ng/ml and run through a sterile filter and stored until use. Insulin (Eli Lilly and Co., USA) stock solutions were similarly prepared in concentrations of 1 U/ml and stored at −20°C until use. Insulin and BMP-4 were added to each well of 12-well plates in a final volume of 1,500 μl.

### 2.2. Morphology and morphometric study

Following insulin and BMP4 treatments, the parasites were microscopically examined daily for 105 days, and the number, size and percentage of microcysts as well as the first days of microcyst formation and deformation were recorded. Also, morphological changes of the microcysts over this time period were evaluated. Microcyst size was measured by using a calibrated microscope. Also, the number of microcysts in flasks was recorded under a light microscope. The percentage of the deformed microcysts was evaluated by measuring total number of microcysts. Hematoxylin and eosin (H&E) staining was also used on formalin-fixed paraffin-embedded sections for morphological comparison between normal and deformed microcysts.

### 2.3. Immunohistochemistry

The samples were fixed and embedded in paraffin before preparing 5-μm cross-sections. Deparaffinized sections were dehydrated by different concentrations of ethanol. The antigen retrieval was performed using citrate buffer (PH = 9.1). The PBS solution was utilized to rinse the cross-sections for 3 min, followed by dipping in hydrogen peroxide (H2O2) for 10 min and 0.3% triton for 30 min to permeate the cell membrane. After washing with PBS, the sections were blocked with 10% goat serum and incubated with primary antibodies including BAX (1:100, SC-7480, Santa Cruz Biotechnology, USA), Bcl-2(1:100, sc-7382 - Santa Cruz Biotechnology, USA). Goat anti-mouse or goat anti-rabbit IgG-CFL 488 (orb688924, Abcam, Cambridge, UK) was used as secondary antibodies for 90 min. Afterwards, the slides were washed and subjected to 4′,6-diamidino-2-phenylindole (DAPI) at an ambient temperature, and re-washed with PBS. A fluorescence microscope equipped with a digital camera was used to take images. All images were captured using the same settings with 200× magnification under a fluorescent microscopy. The quantification of the immunofluorescence assay was based on fluorescence markers. The number of positive cells were counted carefully in 3 sections in 5 fields by a blind investigator. The percentage of positively stained cells (markers) against total nuclei (DAPI) in the regions were evaluated in each group using Image J software v1.8 (NIH, Wayne Rasband, USA) (27).

### 2.4. Alizarin red staining

To evaluate calcification leftovers in the microcysts, the slides were deparaffined and the watering steps were performed. The slides were stained with Alizarin solution (Merck −1.06278) for 1–5 min and observed under light microscope. Calcium red-orange color usually appears in 2 min. To remove excess paint, the slides were first placed in 100% acetone and then in acetone-xylene solution in a one-to-one ratio. After clarification steps, photography was performed with a light microscope (TCM 400, Labomed Inc., CA) (28).

### 2.5. Gene expression analysis

The expressions of BAX, Bcl-2, Caspase-3, Caspase-8 and TNF-α were quantified utilizing real-time PCR (qPCR). The specific primers are shown in Table 1. Total RNA was extracted with TRIzol as described elsewhere (29). The purity and concentrations of RNA were quantified by absorbance measurements at 260/280 nm using NanoDrop 2000 (Nano Drop ND-1000, Nano Drop Technologies, Wilmington, DE). The miscript^®^II Reverse Transcriptase Kit (Qiagen, Germany), was used to construct cDNA and qPCR was performed in a Rotor-Gene Q System (QIAGEN, Hilden, Germany). Thermal cycling conditions were as follows: pre-denaturation at 95°C for 15 min followed by 40 cycles at 95°C for 10 s and 60°C for 34 s. and a final extension of 72°C for 40 s. The relative mRNA expression levels were quantified using the 2^−ΔΔCT^ method.

**Table 1:** Primers used in RT-qPCR for the expression analysis of five genes related to apoptosis in different developmental stages of *Echinococcus granulosus*. 5.8S was used as the reference gene.

**Name**	**Primer**	**Sequence (5' → 3')**
Caspase-3	Forward(f)	CGGCATTTGCTTACCCTATTCAAG
	Reverse(R)	CCTGGATTCCACATCCTGGTAA
Caspase-8	Forward(f)	ATTGGGCAGTGAAGACAAACAAC
	Reverse(R)	TCCAAAGCGAAGGGTGTCTG
BAX	Forward(f)	AAGCATCAACCAATCTGTCAGAG
	Reverse(R)	ACTATCGAAGGAGGCATAAATGTC
Bcl-2	Forward(f)	TCCTCCTCGGATGAAGCAGA
	Reverse(R)	CTGATGTACCGGTCTCTCGC
TNFR	Forward(f)	GCGTTCTATGAGGAGAAGATTCAGT
	Reverse(R)	CGTTATTTCCTCCGCACAGTTC
5.8S	Forward(f)	GTCGATGAAGAGTGCAGCCAAC
	Reverse(R)	GGACAGACGTGACCACAGGC

### 2.6. Molecular dynamics

The sequence of the *E. granulosus insulin receptor* (INSR) and activin receptor type 2 (ACTR) were retrieved from the GenBank, CDS16915 and KAH9286272.1 for INSR and ACTR, respectively. The gene coding for bone morphogenetic protein receptor in *E. granulosus* has not been properly characterized. Therefore to find the bone morphogenetic protein receptors in *E. granulosus* for performing molecular docking and MD simulations, the human bone morphogenetic protein receptor was used with the accession number NP_001195.2 in the NCBI Gene database. Next, in order to identify a similar structure in *E. granulosus*, a protein-protein blast was performed using the blast page against the “non-redundant protein sequence” database and the selected organism *E. granulosus*. Finally, the obtained results were evaluated based on identity, query coverage and e-value.

The crystallographic structure of human insulin in complex with cucurbit (RCSB accession code:3Q6E) was used for docking and molecular dynamics simulations. The water molecules and the ligand were removed from the PDB file and only the two chains of insulin protein remained. The Robetta protein modeling server was utilized to build the ab-initio three-dimensional structure of INSR. Swiss model server was used to predict ACTR. FG-MD server was used for the structure refinement step. The quality and validation of the structure was evaluated by PROSA, Molprobity, Verify3D servers and Ramachandran plot.

To predict the extracellular domain of protein receptors, we used TOPCONS server, a consensus-based server that determines the subcellular location of different parts of a user-defined protein. CASTp server was used to determine the predicted structures' potential binding pocket geometry and amino acids. The HDOCK server was used in the insulin-INSR docking. HDOCK is a hybrid method that consists of template-based modeling and free docking. The structure with the lowest energy was used for further analyses (30).

Different biomolecular structures were simulated by GROMACS 5.0.4 (2021) (31). OPLS-AA forcefields were applied on all the simulations. A dodecahedron box was created, filled with TIP3P water molecules, and neutralized. The structures were energy minimized using a steepest descent algorithm with 50,000 steps and step size of 0.01 kJ/mol/nm. The minimization was terminated when the maximum force <1,000.0 kJ/mol/nm. All the simulations were carried out with a time step of 2fs. The Nose-Hoover thermostat and Parrinello-Rahman pressure coupling were utilized in the system to maintain a constant temperature of 300 K and a constant pressure of 101.3 kPa. Periodic boundary conditions were applied to the system. The energy minimized structure underwent a 50-ps NVT and then a 50-ps NPT ensembles. The equilibrated structure was used for the MD production run. INSR 20-ns MD simulation were carried out to assess the stability of the structure. Then the best posed docked structure of insulin-INSR 20-ns MD simulation was used to assess the binding energy of the complex. End-state free energy calculations were done by Molecular mechanics/Poisson–Boltzmann (Generalized-Born) surface area method using the gmx_MMPBSA tool in GROMACS. Using MDAnalysis package, Root mean square deviation (RMSD), Root mean square fluctuation (RMSF), Radius of gyration, principal component analysis and hydrogen bond analysis was performed.

### 2.7. Statistical analysis

The data are presented as the mean ± standard deviation (SD) and were plotted with GraphPad Prism 8.0 software. Multiple comparisons and within groups were assessed for statistical significance using one-way ANOVA. Values of *P* < 0.05 were considered statistically significant.

## 3. Results

**Figure 2:**
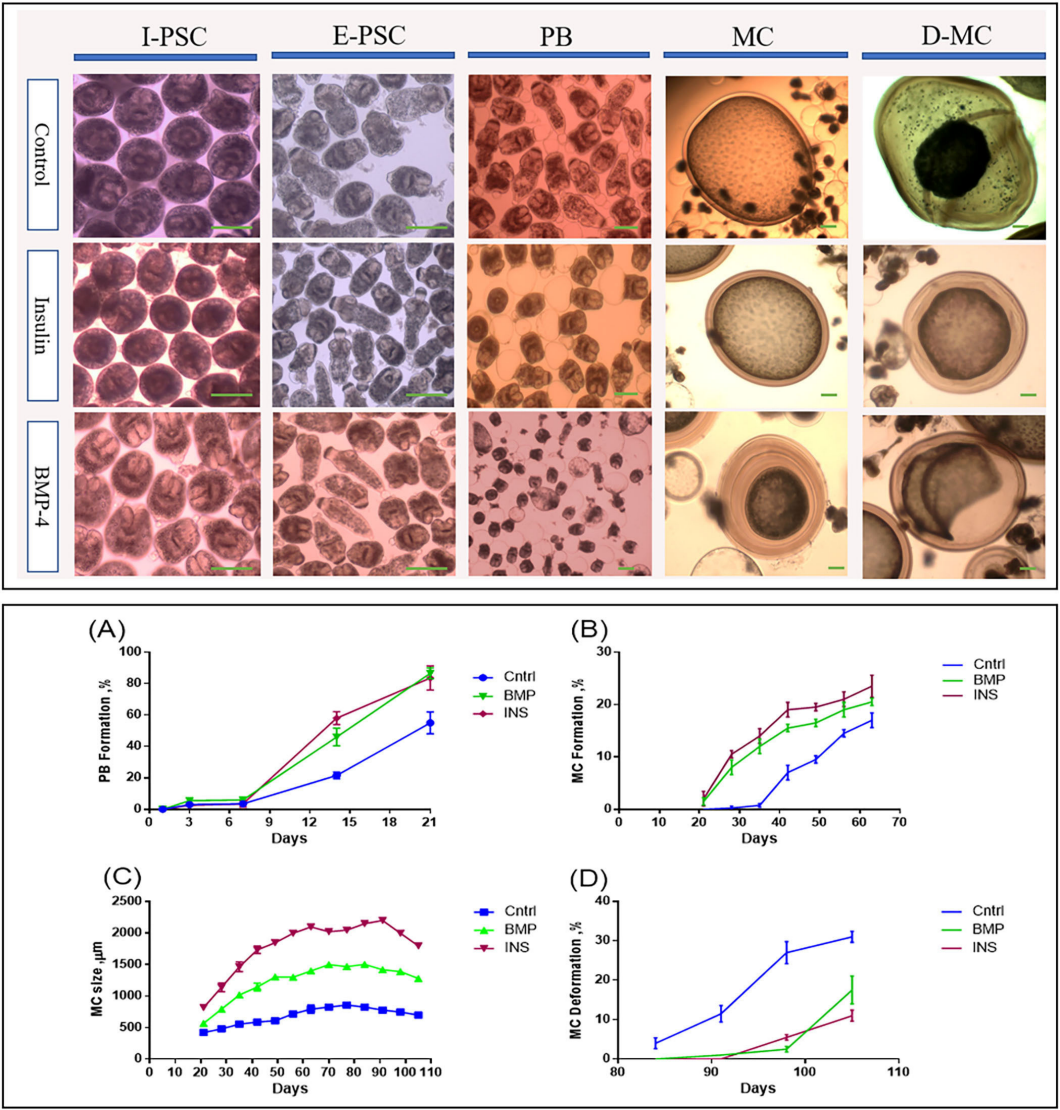
Morphological and developmental changes in different stages of *Echinococcus granulosus* from 2000 protoscoleces cultured in DMEM medium. Upper panel: Morphological changes in different *in vitro* developmental stages of *Echinococcus granulosus* treated with insulin and BMP-4 compared to the control. I-PSC, Invaginated protoscoleces; E-PSC, Evaginated protoscoleces; PB, Posterior Bladder formation; MC, Microcysts; D-MC, Deformed Microcysts. Lower panel: Developmental changes induced by insulin and BMP-4 treatment in different *in vitro* stages of *Echinococcus granulosus*. **(A)** Posterior Bladder (PB) formation rate. **(B)** Microcyst formation rate. **(C)** Average size changes of the microcysts. **(D)** Microcyst deformation rate. Scale bar: 200 μm.

Figure 2 shows the developmental stages of *E. granulosus* sensu stricto G1 genotype (accession No. OP882698) in the monophasic medium comparing the invaginated PSCs (I-PSC), evaginated PSCs (E-PSC), protoscoleces with posterior bladder (PB), mature microcysts (MC) and deformed microcysts (D-MC) in insulin / BMP4 treated groups as well as the control group.

From day 7 onwards, bladder-like vesicles known as posterior bladders were appeared at the posterior end of the protoscoleces in all groups. The percentages of PB formation in the PSCs treated with insulin and BMP-4 were significantly increased compared to the control PSCs, so that at the end of day 21, PB formation in the PSCs was 55, 83.5, and 86.5% in the control, insulin and BMP-4 respectively (Figure 2A). While in the control group, the first microcyst was found on day 28, in the insulin and BMP groups the first microcysts were formed on day 21. On the day 63, while 17% of microcysts were present in the control group, nearly 23% of microcysts were formed in the insulin group and 20% in the BMP group (Figure 2B).

**Figure 3:**
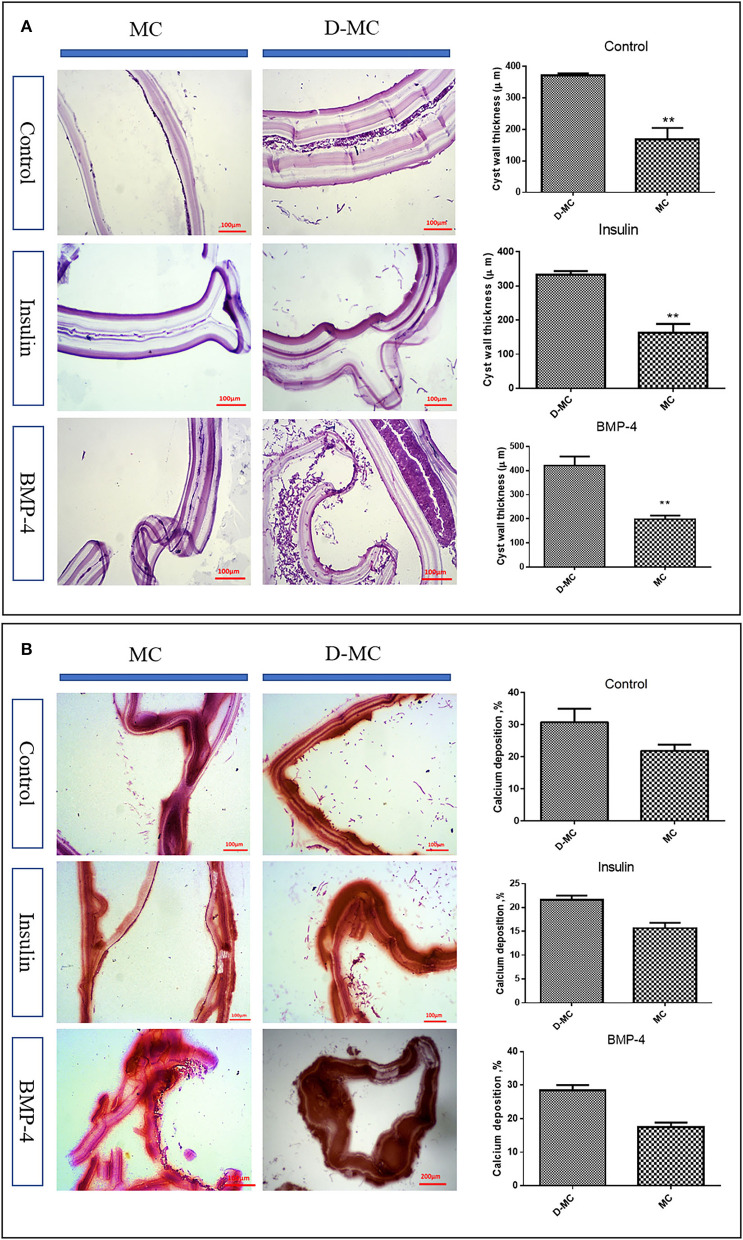
*In vitro* histological changes in the microcysts of *Echinococcus granulosus* following treatment with insulin and BMP-4. **(A)** Changes in the laminated layer (LL) thickness of the microcysts of *Echinococcus granulosus* following treatment with insulin and BMP-4, Hematoxylin and Eosin (H&E) staining. MC, Microcyst; D-MC, Deformed microcyst. Bars show the mean±standard deviation (SD). **P < 0.005. **(B)** Calcium deposition changes in the microcysts of *Echinococcus granulosus* treated with insulin and BMP-4, Alizarin red staining. MC, Microcyst; D-MC, Deformed Microcyst.

According to Figure 2C, the average size of microcysts in the insulin and BMP groups was significantly greater than the control group, and the size began to decrease from the 84th day. There are very little variations in the cysts size within each group, however the insulin and BMP-4 treated groups produced significantly larger microcysts compared to the control group. Also within insulin and BMP-4 groups, we did not observe any significant variations at different time points. As shown in Figure 2D, in the control group, from day 84, the microcysts began to wrinkle so that the germinal layer was partially detached from the laminated layer. In the control group a higher percentage of microcysts were deformed by the end of the day 105 and this deformation occurred earlier than the groups treated with insulin and BMP-4. On the other hand, H&E staining showed (Figure 3A) that in all groups the thickness of the laminated layer in the wrinkled microcysts was greater than the normal microcysts with the same age (*p* < 0.05). Alizarin Red staining of the microcysts indicates that calcium accumulation in deformed microcysts is higher than the normal ones. Notably the intensity of calcium deposition in the insulin and BMP groups was lower than the control group (Figure 3B).

**Figure 4:**
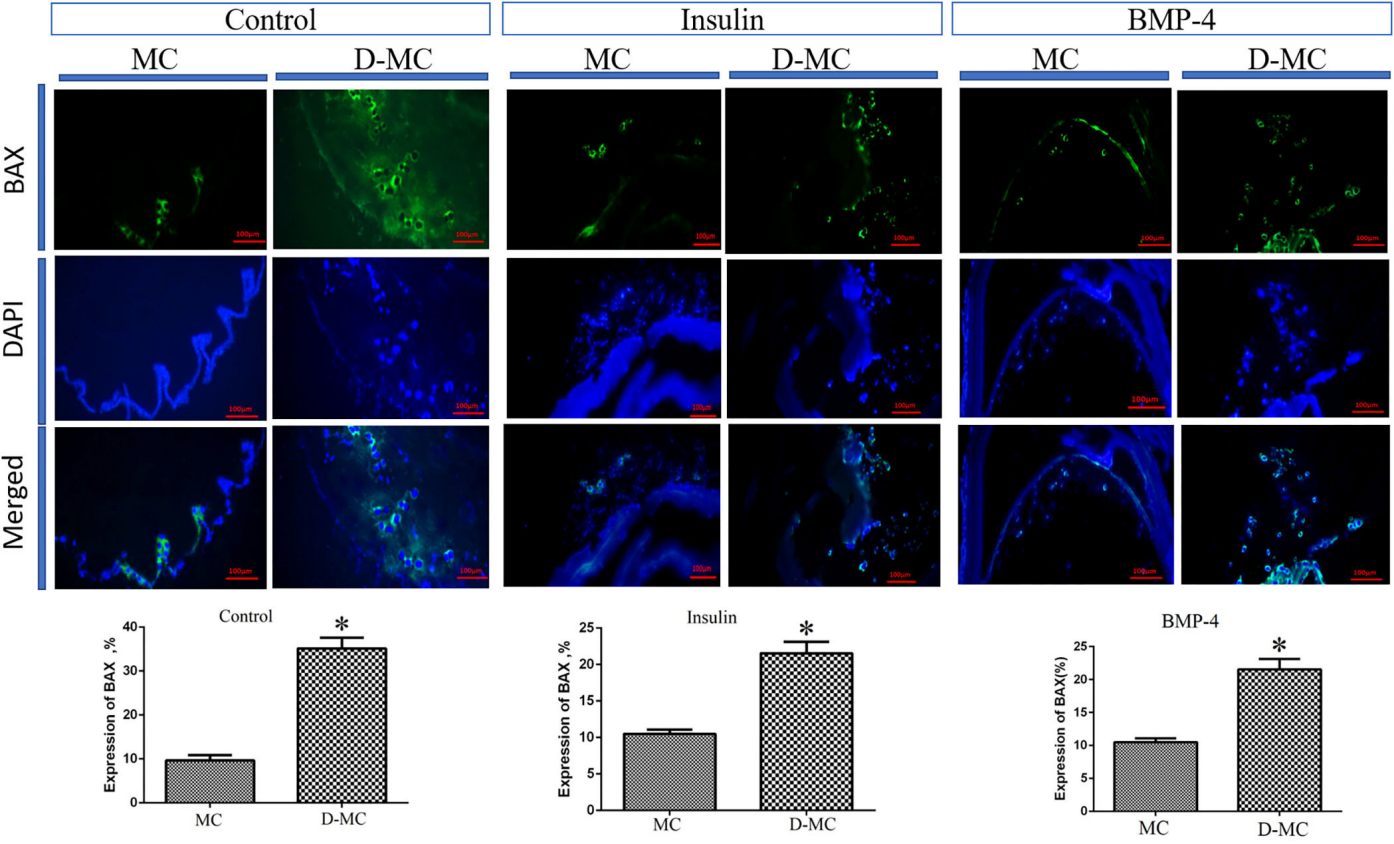
BAX expression changes in microcysts of *Echinococcus granulosus* treated *in vitro* with insulin and BMP-4 compared to the control, immunohistochemistry staining. MC, Microcyst; D-MC, Deformed Microcyst. (*P < 0.05).

**Figure 5:**
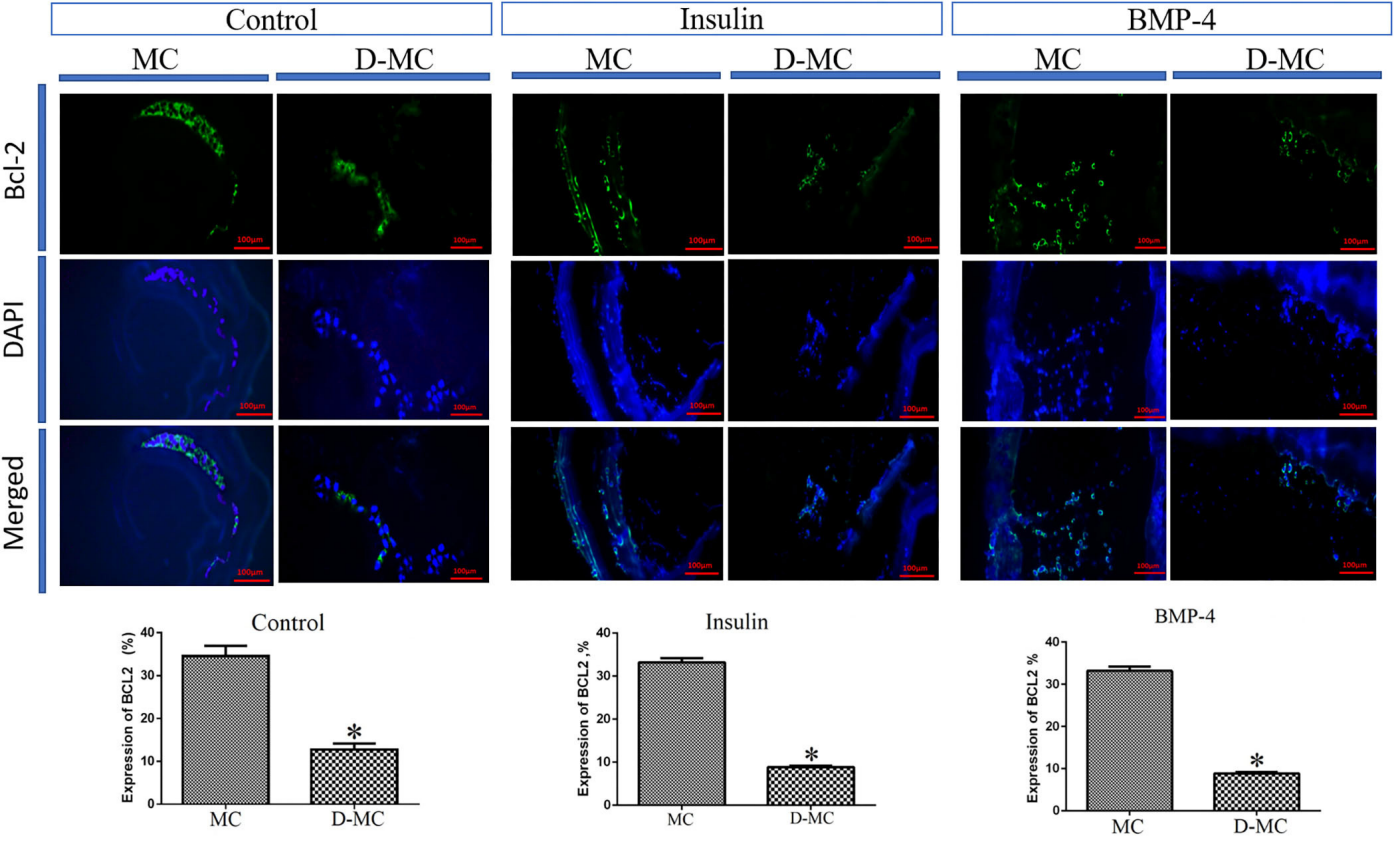
Bcl-2 expression changes in microcysts of *Echinococcus granulosus* treated *in vitro* with insulin and BMP-4 compared to the control, immunohistochemistry staining. MC, Microcyst; D-MC, Deformed Microcyst. (*P < 0.05).

The comparison of IHC in different groups showed BAX expression is significantly higher in the deformed microcysts than in the normal ones. Similar findings were also obtained in the groups treated with insulin and BMP-4, but BAX expressions in these two groups were less than the control parasites (Figure 4). IHC analysis of Bcl-2indicates a decreased expression in deformed microcysts compared to the intact microcysts in all experimental groups. Notably Bcl-2 expression was lower in the insulin and BMP-4 treatments compared to the control (Figure 5).

**Figure 6:**
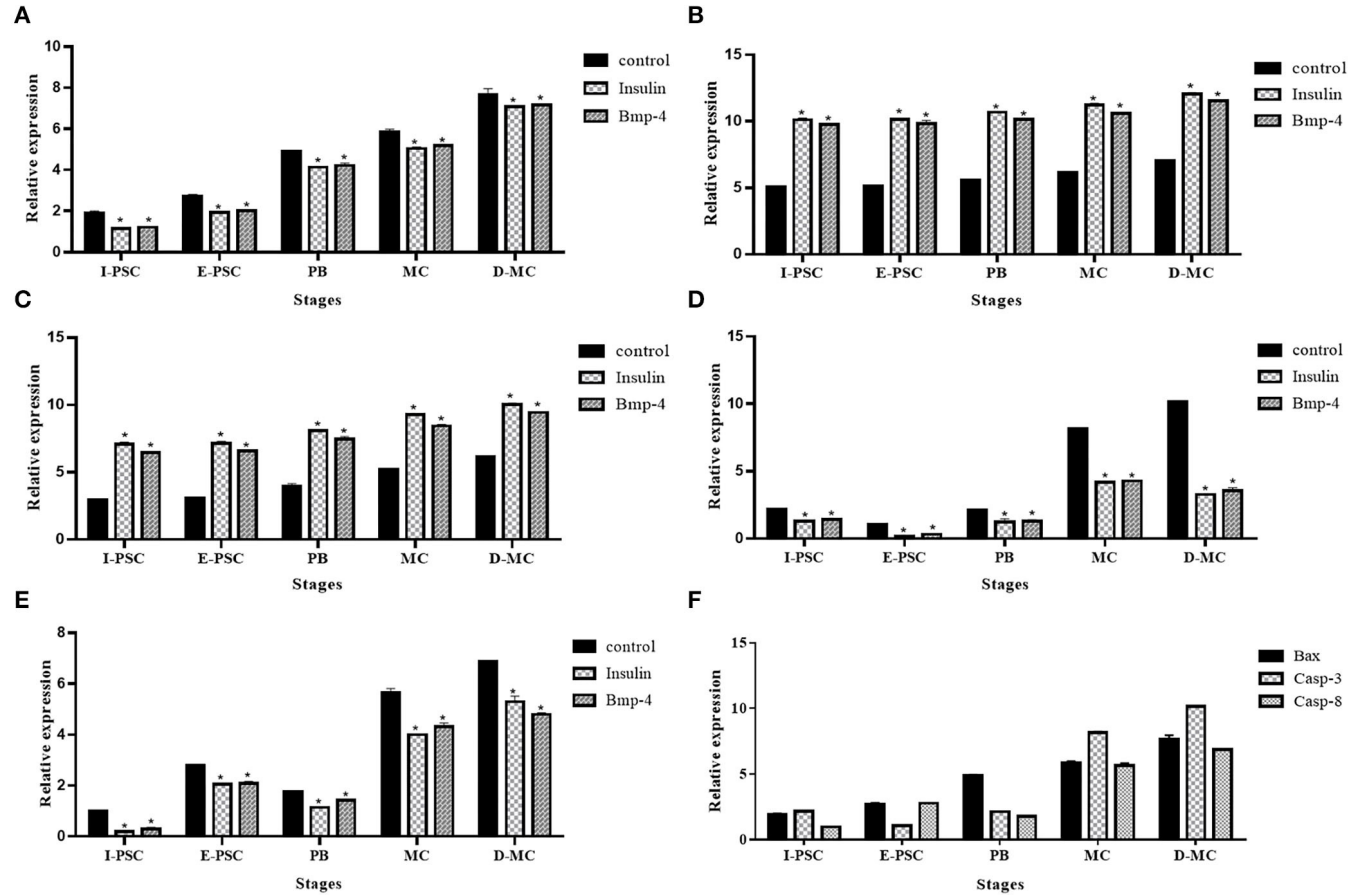
Expression profile of five genes, **(A)** BAX, **(B)** Bcl-2, **(C)** Caspase-3, **(D)** Caspase-8, **(E)** TNF-α, related to apoptosis in different *in vitro* developmental stages of *Echinococcus granulosus* treated with insulin and BMP-4 compared to the control using RT-qPCR. I-PSC, Invaginated protoscoleces; E-PSC, Evaginated protoscoleces; PB, Posterior Bladder; MC, Microcyst; D-MC, Deformed Microcyst. Bars show the mean ± standard deviation (SD) derived from duplicate experiments. **(F)** pro-apoptosis genes in control group (*P < 0.05).

Figure 6 shows the expression profiles of the five genes, including BAX, Bcl-2, TNF-α, Caspas-3 and Caspase-8, involved in apoptotic changes in different *in vitro* developmental stages of *E. granulosus*, in the control, as well as insulin and BMP-4 treatment groups.

The expression of BAX, Caspase-3 and Caspase-8 represented a significant increase in the control group compared to insulin and BMP-4. In contrast, a significant decrease in Bcl-2 and TNF-α expression were observed in controls compared to insulin and BMP-4 groups. As shown in Figure 6F, deformed microcysts presented significant apoptotic changes compared to other stages of parasite development *in vitro* in the control groups.

Molecular dynamic studies indicate that BMPs possess a wide range of biological activities in different tissues. These proteins are members of the transforming growth factor β (TGF-β) family, which bind to serine-threonine kinase type II and type I receptors. The results of protein-protein blast showed that human bone morphogenetic protein receptors has the most similarity to the parasite Activin receptor type-2A with accession number KAH9286272.1. Therefore, this sequence was used to determine the 3D structure, docking and MD simulations.

INSR has a substantial, large three-dimensional structure. Due to the sparsity of protein structure data in *E. granulosus*, the prediction quality would be greatly deranged because of protein size. Also, molecular dynamics simulations would be time-consuming, and the probability of trapping in a local minimum is proportionally increased. Thus, the TOPCONS server has been used for the identification of the extracellular part of the insulin receptor. The consensus prediction of the TOPCONS server was used as the concluding prediction of the subcellular localization of the receptor. Of the 1,740 amino acids, 1,037 amino acids are extracellular, 20 and 616 residues were transmembrane and intracellular, respectively. Robetta server, an enhanced version of alpha fold 2, has been used to predict the three-dimensional structure of the extracellular part of INSR. Then the FG_MD server was utilized for the structure refinement step. The best of the five predicted structures has an overall quality factor of 83.8552 (ERRAT score); 79.75% of the residues have averaged a 3D-1D score ≥0.2 (VERIFY 3D), PROSA z score of −11.18 and MolProbity score of 2.57. Ramachandran plots of the proteins are shown in Supplementary Figure 1. In the case of ACTR, the whole protein sequence has been utilized for modeling using the Swiss model server, and the final predicted structure achieved 94. 1176 ERRAT score, 80.66% (Verify 3d), z-score of −8.55 (PROSA), and molprobity score of 1.66.

**Figure 7:**
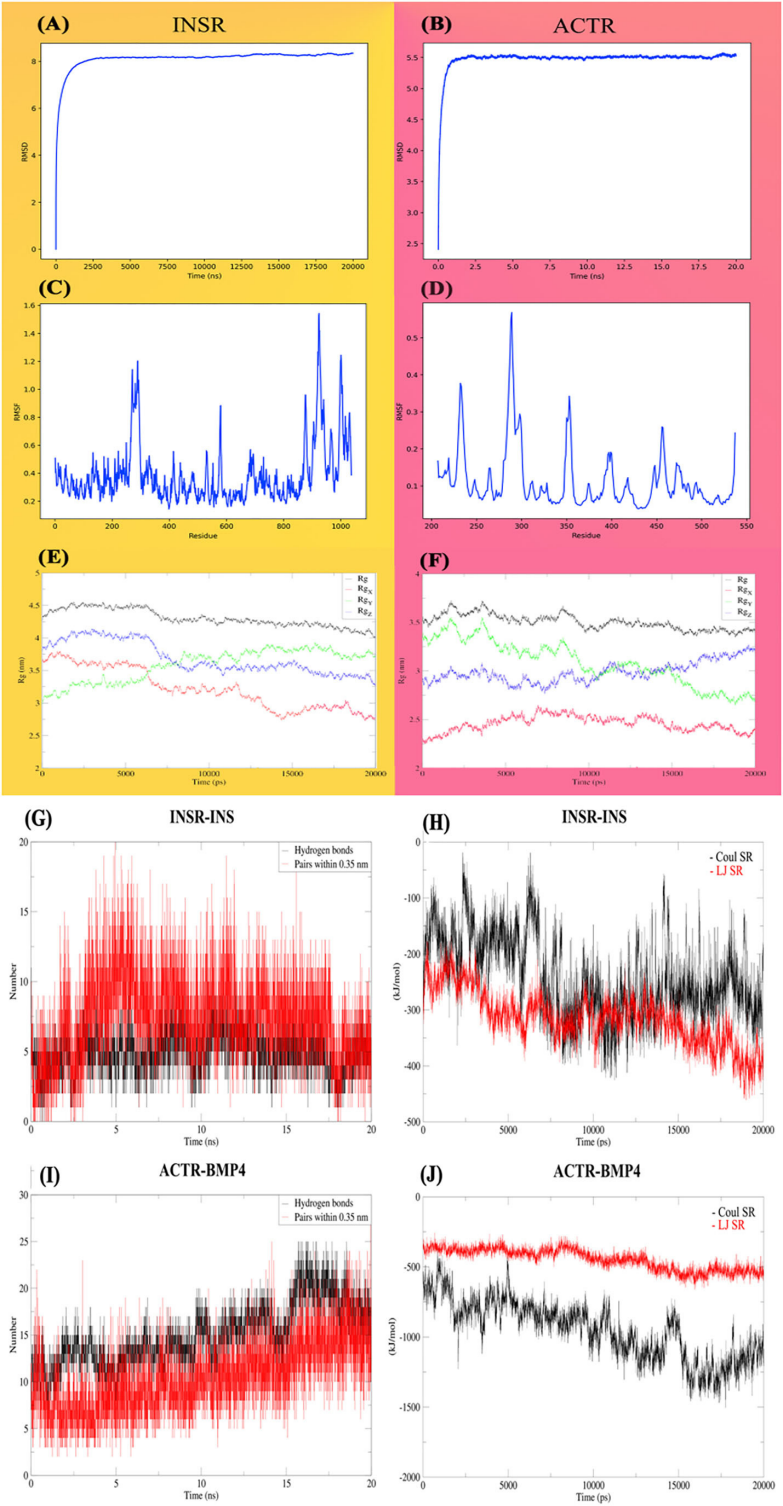
Molecular dynamic analysis of the stability and interactions of INSR and ACTR receptors with their corresponding ligands. INSR and ACTR total and around the axes plots, RMSD **(A, B)**, RMSF **(C, D)**, and radius of gyration (Rg) **(E, F)**. Hydrogen bonds **(G, H)** and energies of interaction **(I, J)**, Coulomb, Lennard-Jones and, short-range energies.

The First 20-ns MD simulation, which is solely based on receptor structures, showed that the predicted structures preserve a relatively stable form according to the RMSD, depicted in Figures 7A, B. Both of the receptors reached a plateau after a few ns, and the RMSD value remains constant. Likewise, for most of the INSR amino acid residues, RMSF values are <1 Angstrom (Figures 7C, D) for most of the residues; however, the residues placed in the binding pocket had the most fluctuations. Another indicator of stability is the radius of gyration which tries to quantify the tendency of a protein for compactness. The mean radius of gyration for INSR was 4.30 (95% CI of ± 0.0026); it was 3.50 (95% CI of ± 0.0015) which overall denotes the stability of the receptor structures (Figures 7E, F). In case of energies of interactions receptors showed a strong propensity for interaction with their corresponding ligands (Figures 7G, H). The fluctuation of hydrogen bonds during the simulations are depicted in Figures 7I, J.

## 4. Discussion

Currently, our knowledge on the natural history of cystic echinococcosis, and the cyst evolution over time, is limited. In a clinical context the World Health Organization classified hepatic cysts into 6 stages from a single well-defined unilocular hydatid cyst (CE1) to the cysts with detached laminated/germinal membranes (CE3a), and finally to the fully calcified degenerated hydatid cysts (CE5) (32). Studies have shown that *E. granulosus* cysts can survive in the liver with no specific symptoms for years (33, 34). However, there are still many questions on the process in which these stages are evolved, how one stage transforms into the another, what host- or environmentally related factors affecting these changes and the molecular and ultrastructural characteristics of this process both in *in vivo* and *in vitro* settings. Many studies have been carried out on the bi-directional development of *E. granulosus* in mono- and d-phasic culture systems, however our understanding on the natural history of microcysts in prolonged *in vitro* culture and molecular events during this period is very limited.

Our findings on the molecular and histological markers of apoptosis, calcification and degeneration provide significant evidence suggesting that the microcysts cultivated *in vitro* can undergo processes similar to what have been observed in the natural history of hydatid cysts in *in vivo* settings i.e. in humans and certain intermediate hosts. The findings of our study showed that after 84 days, the germinal layer of the microcysts wrinkled and started to separate from the laminated layer. Kern et al. showed that a percentage of hydatid cysts undergo spontaneous physical changes over time, including separation of the cyst membranes, rupture of daughter cysts, and other signs of degeneration and loss of viability (33, 34). Also, our findings indicate that calcium accumulation in deformed microcysts on day 105 of parasite cultivation is higher compared to the normal microcysts (Figure 3B). The significant changes in BAX and Bcl-2 expression in the deformed microcysts compared to normal ones, is suggestive of apoptotic processes in the prolonged microcyst culture *in vitro*. It could therefore be assumed that hydatid cyst calcification changes occur in the absence of host-related factors.

The influence of growth factors is also shown in this study. Our findings showed that apoptosis and deformation of the microcysts can be delayed under the influence of insulin and BMP-4, indicating that cellular factors and host hormones can help in increasing the lifespan of hydatid cysts. Helminths are multicellular organisms that require cell-to-cell communication throughout their growth and development. The event of cell signaling determines a cell's fate, organizes tissues, establishes a body plan, and initiates and maintains the process of reproductive reproduction (35). Hormones and cytokines such as insulin and BMP-4 play a significant role in the development, differentiation and apoptosis of the helminths. Although the role of insulin and BMP-4 have been extensively studied and well characterized in several helminth parasites, including *E. multilocularis* (15, 16, 23), *Schistosoma mansoni* (17, 18, 22, 36) and *S. japonicum* (19, 37), there are no published data on the effect of insulin and BMP-4 on the biological properties of *E. granulosus*. In the current study, we investigated the effects of the growth factors, insulin and BMP-4, on the growth and development of *E. granulosus*. In general, the groups treated with insulin and BMP-4 produced higher number of microcysts and PSCs with posterior bladder, while there were no significant differences between the insulin and BMP groups (Figures 2A, B). The posterior bladders appeared at the posterior end of some of the protoscoleces in all groups. The material of which these fluid-filled posterior bladders are composed, appears to be continuous with the tegument covering the posterior regions of the protoscoleces and can grow to relatively large size. As shown in Figure 2 both evaginated and invaginated protoscoleces in monophasic media can develop into microcysts through the posterior bladders. Also the findings documented a larger size of the microcysts and a delay in the process of apoptosis and aging of the microcysts. We also showed that calcium deposition in microcysts treated with the insulin and BMP-4 is lower than the untreated microcysts, indicating that the calcification process in microcysts treated with the insulin and BMP-4 was slowed down.

In line with our data Hemer et al. found an almost sixfold increase in *E. multilocularis* vesicle formation after 1 week of incubation of primary cells with insulin. Also, they documented a 50% increase in microcyst formation following 3 weeks of *in vitro* culture of the protoscoleces with insulin (16). Also, another study showed that host-derived EGFs can promote germinative cell proliferation in *E. multilocularis* and may interfere with the TGF-β/BMP family in the metacestode (38).

Our findings indicate that treatment with insulin and BMP-4 led to the increased average size of microcysts compared to the control groups (Figure 2C). Other studies linked the effects of insulin and BMP with the glucose metabolism, growth and development in other helminth species including Mesocestoides corti (39) and Taenia crassiceps [(40, 41) #30]. Adalid- Peralta et al. showed that host-TGF- β interacts with the parasite TGF-β/BMP receptors to enhance the growth of Taenia crassiceps metacestodes (41). In E. multilocularis (16) and S. mansoni (19) it has been shown that using insulin receptor inhibitors can disrupt the growth and development of the parasite. The inhibitors induced significant effects on the re-differentiation process of E. multilocularis protoscoleces toward the metacestode stage (16). In another study it was shown that RTK inhibitors can induce apoptosis in S. mansoni, by inhibiting the insulin receptor and tyrosine kinases (42). Molecular dynamics findings of our study largely support the hypothesis that the parasite can be nourished by human growth factors.

In the current study, IHC was performed to evaluate the process of apoptosis in the normal microcysts, deformed microcysts, and the microcysts treated with insulin and BMP-4. Our results showed a significant decrease in BAX and an increase in Bcl-2 in deformed microcysts, which is an indication of decreased apoptosis compared to the untreated groups. IHC findings are in line with our RT-qPCR data of the expression of apoptosis-related genes including Caspase-3, Caspase-8, TNF-α, BAX and Bcl-2. It can be clearly understood that the microcysts exposed to insulin and BMP are less affected by apoptotic events as revealed by the increase in BAX, Caspase-3 and Caspase-8 genes expression as well as the decrease in TNF-α and Bcl-2 expression in the normal microcysts compared to the insulin and BMP groups (Figure 6). It has been determined that an increase in the expression of apoptosis-inducing ligands, caspase 3 and DNA fragmentation occurs on the surface of the germinal layer of infertile cysts compared to the fertile cysts and host tissues, which indicate that apoptotic events can play an important role in the natural history of cystic echinococcosis (43, 44). However our knowledge is very limited on the apoptotic events in *E. granulosus* during *in vitro* development based on a stage-specific approach over time for more than 100 days. Most of the *in vitro* and *in vivo* studies has only evaluated apoptosis in the microcysts and protoscoleces following exposure to certain chemotherapeutic agents including dexamethasone, praziquantel, albendazole sulfoxide and nano compounds (45–49).

It should be noted that in the *in vivo* setting the biology of host-parasite interplay is more complex, therefore, the interpretation of the results of *in vitro* observations should be made with caution. Further studies on host-parasite interactions and the effects of hormones and cytokines on *E. granulosus* cyst development in different intermediate hosts are required at the level of signaling pathways, insulin and BMP inhibitors, other BMP classes and transcriptome level studies. Experimental studies are also required to investigate the outcomes of insulin and BMP receptors blocking on *E. granulosus* growth and development.

Findings of the present study provide evidences of calcification of the microcysts of *E. granulosus* in a long term *in vitro* cultivation as revealed by the significant increase in apoptotic events and calcification-specific staining methods. Also the study conclude that insulin and BMP-4 delay the process of microcyst deformation, apoptosis and calcification. The results present important implications for improving our knowledge on the nature of hydatid cyst calcification in the hosts and provide a potential for designing and development of drugs.

## Data availability statement

The original contributions presented in the study are included in the article/Supplementary material, further inquiries can be directed to the corresponding author.

## Author contributions

AD, SM, and MF: conceptualization, study design, data validation, and writing–original draft preparation. AD and SM: data curation and laboratory experiments. AD, AK, SM, and MF: data analysis. AD, MR, AA, and MF: molecular dynamics. MF: funding acquisition. AD, SM, MR, AA, MM, SD, and MF: revising and final approval of the manuscript. All authors contributed to the article and approved the submitted version.

## References

[1] Craig PS McManus DP Lightowlers MW Chabalgoity JA Garcia HH Gavidia CM. Prevention and control of cystic echinococcosis. Lancet Infect Dis. (2007) 7:385–94. 10.1016/S1473-3099(07)70134-2 17521591

[2] Deplazes P Rinaldi L Rojas CA Torgerson P Harandi M Romig T. Global distribution of alveolar and cystic echinococcosis. Adv Parasitol. (2017) 95:315–493. 10.1016/bs.apar.2016.11.001 28131365

[3] Thompson R. Biology and systematics of echinococcus. Adv Parasitol. (2017) 95:65–109. 10.1016/bs.apar.2016.07.001 28131366

[4] Rogan M Bodell A Craig P. Post-encystment/established immunity in cystic echinococcosis: is it really that simple? Parasite Immunol. (2015) 37:1–9. 10.1111/pim.12149 25283301

[5] Smyth J. Studies on tapeworm physiology: XI. *In vitro* cultivation of *Echinococcus granulosus* from the protoscolex to the strobilate stage. Parasitology. (1967) 57:111–33. 10.1017/S0031182000071936

[6] Smyth J Howkins A Barton M. Factors controlling the differentiation of the hydatid organism, *Echinococcus granulosus*, into cystic or strobilar stages *in vitro*. Nature. (1966) 211:1374–7. 10.1038/2111374a0 5969832

[7] Thompson R Jenkins D. Echinococcus as a model system: biology and epidemiology. Int J Parasitol. (2014) 44:865–77. 10.1016/j.ijpara.2014.07.005 25123067

[8] Brehm K Koziol U. Echinococcus–host interactions at cellular and molecular levels. Adv Parasitol. (2017) 95:147–212. 10.1016/bs.apar.2016.09.001 28131363

[9] Brehm K. The role of evolutionarily conserved signalling systems in Echinococcus multilocularis development and host-parasite interaction. Med Microbiol Immunol. (2010) 199:247–59. 10.1007/s00430-010-0154-1 20376483

[10] Brehm K. Echinococcus multilocularis as an experimental model in stem cell research and molecular host-parasite interaction. Parasitol. (2010) 137:537–55. 10.1017/S0031182009991727 19961652

[11] Pires-daSilva A Sommer RJ. The evolution of signalling pathways in animal development. Nat Rev Genet. (2003) 4:39–49. 10.1038/nrg977 12509752

[12] Gems D Partridge L. Insulin/IGF signalling and ageing: seeing the bigger picture. Curr Opin Genet Dev. (2001) 11:287–92. 10.1016/S0959-437X(00)00192-1 11377965

[13] Kaletsky R Murphy CT. The role of insulin/IGF-like signaling in C. elegans longevity and aging. Dis Models Mech. (2010) 3:415–9. 10.1242/dmm.001040 20354111

[14] Teleman AA. Molecular mechanisms of metabolic regulation by insulin in Drosophila. Biochem J. (2010) 425:13–26. 10.1042/BJ20091181 20001959

[15] Konrad C Kroner A Spiliotis M Zavala-Góngora R Brehm K. Identification and molecular characterisation of a gene encoding a member of the insulin receptor family in Echinococcus multilocularis. Int J Parasitol. (2003) 33:301–12. 10.1016/S0020-7519(02)00265-5 12670515

[16] Hemer S Konrad C Spiliotis M Koziol U Schaack D Förster S. Host insulin stimulates Echinococcus multilocularisinsulin signalling pathways and larval development. BMC Biol. (2014) 12:1–22. 10.1186/1741-7007-12-5 24468049 PMC3923246

[17] Ahier A Khayath N Vicogne J Dissous C. Insulin receptors and glucose uptake in the human parasite Schistosoma mansoni. Parasite. (2008) 15:573–9. 10.1051/parasite/2008154573 19202764

[18] Khayath N Vicogne J Ahier A BenYounes A Konrad C Trolet J. Diversification of the insulin receptor family in the helminth parasite Schistosoma mansoni. FEBS J. (2007) 274:659–76. 10.1111/j.1742-4658.2006.05610.x 17181541

[19] You H Zhang W Jones MK Gobert GN Mulvenna J Rees G. Cloning and characterisation of Schistosoma japonicum insulin receptors. PLoS ONE. (2010) 5:e9868. 10.1371/journal.pone.0009868 20352052 PMC2844434

[20] Du X Jones MK Nawaratna SS Ranasinghe S Xiong C Cai P. Gene expression in developmental stages of Schistosoma japonicum provides further insight into the importance of the Schistosome insulin-like peptide. Int J Mol Sci. (2019) 20:1565. 10.3390/ijms20071565 30925781 PMC6480100

[21] Xiao YT Xiang LX Shao JZ. Bone morphogenetic protein. Biochem Biophys Res Commun. (2007) 362:550–3. 10.1016/j.bbrc.2007.08.045 17719560

[22] Freitas TC Jung E Pearce EJ. A bone morphogenetic protein homologue in the parasitic flatworm, Schistosoma mansoni. Int J Parasitol. (2009) 39:281–7. 10.1016/j.ijpara.2008.08.001 18765241 PMC2852128

[23] Epping K Brehm K. Echinococcus multilocularis: molecular characterization of EmSmadE, a novel BR-Smad involved in TGF-β and BMP signaling. Exp Parasitol. (2011) 129:85–94. 10.1016/j.exppara.2011.07.013 21802416

[24] Zavala-Gongora R Kroner A Bernthaler P Knaus P Brehm K A. member of the transforming growth factor-ß receptor family from Echinococcus multilocularis is activated by human bone morphogenetic protein 2. Mol Biochem Parasitol. (2006) 146:265–71. 10.1016/j.molbiopara.2005.12.011 16434111

[25] Smyth J Davies Z. *In vitro* culture of the strobilar stage of *Echinococcus granulosus* (sheep strain): a review of basic problems and results. Int J Parasitol. (1974) 4:631–44. 10.1016/0020-7519(74)90028-9 4609935

[26] Mousavi SM Afgar A Mohammadi MA Mortezaei S Sadeghi B Harandi MF. Calmodulin-specific small interfering RNA induces consistent expression suppression and morphological changes in *Echinococcus granulosus*. Sci Rep. (2019) 9:1–9. 10.1038/s41598-019-40656-w 30846822 PMC6406006

[27] Burry RW. Immunocytochemistry. Springer Sci Bus Media. (2009) 10:978–1. 10.1007/978-1-4419-1304-3_1

[28] Gregory CA Gunn WG Peister A Prockop DJ. An Alizarin red-based assay of mineralization by adherent cells in culture: comparison with cetylpyridinium chloride extraction. Anal Biochem. (2004) 329:77–84. 10.1016/j.ab.2004.02.002 15136169

[29] Faridi A Mansouri M Macchiaroli N Afgar A Mousavi SM Rosenzvit MC. Harandi, MicroRNA profile of the strobilated worms of Echinococcus granulosus derived from *in vivo* and *in vitro* systems by using high-throughput approach. Parasitol Res. (2021) 120:3203–14. 34351489 10.1007/s00436-021-07251-3

[30] Yan Y Zhang D Zhou P Li B Huang SY. HDOCK a web server for protein–protein and protein–DNA/RNA docking based on a hybrid strategy. Nucleic Acids Res. (2017) 45:W365–W73. 10.1093/nar/gkx407 28521030 PMC5793843

[31] Abraham MJ Murtola T Schulz R Páll S Smith JC Hess B. GROMACS: High performance molecular simulations through multi-level parallelism from laptops to supercomputers. SoftwareX. (2015) 1:19–25. 10.1016/j.softx.2015.06.001

[32] Group WIW. International classification of ultrasound images in cystic echinococcosis for application in clinical and field epidemiological settings. Acta Trop. (2003) 85:253–61. 10.1016/S0001-706X(02)00223-1 12606104

[33] Kern P. Echinococcus granulosus infection: clinical presentation, medical treatment and outcome. Langenbeck's Arch Surg. (2003) 388:413–20. 10.1007/s00423-003-0418-y 14605887

[34] Solomon N Kachani M Zeyhle E Macpherson C. The natural history of cystic echinococcosis in untreated and albendazole-treated patients. Acta Trop. (2017) 171:52–7. 10.1016/j.actatropica.2017.03.018 28336270

[35] Castro GA. Helminths: structure, classification. growth, and development flukes (trematodes). In:Baron S, editor. Medical Microbiology. 4th ed. Galveston, TX: University of Texas Medical Branch at Galveston (1996). 21413320

[36] Liu R Zhao QP Ye Q Xiong T Tang CL Dong Hf. Cloning and characterization of a bone morphogenetic protein homologue of Schistosoma japonicum. Exp Parasitol. (2013) 135:64–71. 10.1016/j.exppara.2013.05.016 23756146

[37] Du X McManus DP Cai P Hu W You H. Identification and functional characterisation of a Schistosoma japonicum insulin-like peptide. Parasit Vectors. (2017) 10:1–12. 10.1186/s13071-017-2095-7 28407789 PMC5391603

[38] Cheng Z Liu F Li X Dai M Wu J Guo X. EGF-mediated EGFR/ERK signaling pathway promotes germinative cell proliferation in Echinococcus multilocularis that contributes to larval growth and development. PLoS Negl Trop Dis. (2017) 11:e0005418. 10.1371/journal.pntd.0005418 28241017 PMC5344531

[39] Canclini L Esteves A. *In vivo* response of Mesocestoides vogae to human insulin. Parasitology. (2009) 136:203–9. 10.1017/S003118200800526X 19079819

[40] Escobedo G Romano M Morales-Montor J. Differential *in vitro* effects of insulin on Taenia crassiceps and Taenia solium cysticerci. J Helminthol. (2009) 83:403–12. 10.1017/S0022149X09990265 19549345

[41] Adalid-Peralta L Rosas G Arce-Sillas A Bobes RJ Cárdenas G Hernández M. Effect of transforming growth factor-β upon Taenia solium and Taenia crassiceps cysticerci. Sci Rep. (2017) 7:1–13. 10.1038/s41598-017-12202-z 28955045 PMC5617888

[42] Vanderstraete M Gouignard N Cailliau K Morel M Lancelot J Bodart J-F. Dual targeting of insulin and venus kinase receptors of Schistosoma mansoni for novel anti-schistosome therapy. PLoS Negl Trop Dis. (2013) 7:e2226. 10.1371/journal.pntd.0002226 23696913 PMC3656120

[43] Spotin A Majdi MMA Sankian M Varasteh A. The study of apoptotic bifunctional effects in relationship between host and parasite in cystic echinococcosis: a new approach to suppression and survival of hydatid cyst. Parasitol Res. (2012) 110:1979–84. 10.1007/s00436-011-2726-4 22167369

[44] Paredes R Jimenez V Cabrera G Iragüen D Galanti N. Apoptosis as a possible mechanism of infertility in *Echinococcus granulosus* hydatid cysts. J Cell Biochem. (2007) 100:1200–9. 10.1002/jcb.21108 17031852

[45] Hu H Kang J Chen R Mamuti W Wu G Yuan W. Drug-induced apoptosis of *Echinococcus granulosus* protoscoleces. Parasitol Res. (2011) 109:453–9. 10.1007/s00436-011-2276-9 21365454

[46] Xing G Wang B Lei Y Liu C Wang Z Shi H. *In vitro* effect of sodium arsenite on *Echinococcus granulosus* protoscoleces. Mol Biochem Parasitol. (2016) 207:49–55. 10.1016/j.molbiopara.2016.05.011 27234209

[47] Naseri M Akbarzadeh A Spotin A Akbari NAR Mahami-Oskouei M Ahmadpour E. Scolicidal and apoptotic activities of albendazole sulfoxide and albendazole sulfoxide-loaded PLGA-PEG as a novel nanopolymeric particle against *Echinococcus granulosus* protoscoleces. Parasitol Res. (2016) 115:4595–603. 10.1007/s00436-016-5250-8 27623699

[48] Shi H Lei Y Wang B Wang Z Xing G Lv H. Protoscolicidal effects of chenodeoxycholic acid on protoscoleces of *Echinococcus granulosus*. Exp Parasitol. (2016) 167:76–82. 10.1016/j.exppara.2016.05.004 27207732

[49] Kang JF Hu HH Yuan WM Wu GZ Chen R Baishanbieke W. Apoptosis induced *in vitro* by dexamethasone and ATP in the protoscolex of *Echinococcus granulosus*. Zhongguo ji Sheng Chong xue yu ji Sheng Chong Bing za zhi=Chin J Parasitol Parasitic Dis. (2009) 27:332–5. 20066991

