# Supplementary material for: Natural history of Echinococcus granulosus microcyst development in long term in vitro culture and molecular and morphological changes induced by insulin and BMP-4
Source: Front Vet Sci. 2023 Jan 9;9:1068602. doi: 10.3389/fvets.2022.1068602 (PMC9868913; doi:10.3389/fvets.2022.1068602)

**Supplementary Figure 1**. The Ramachandran plot for parasitic receptors; Insulin receptor (INSR); Activin receptor type 2 (ACTR). The most favored, additionally allowed, generously allowed and disallowed regions are shown in dark gray, medium gray, pale gray, and white colors, respectively. Triplets of numbers on the bottom left on each plot indicate, from top to bottom: percentage of residues in highly favored, favored, and questionable regions respectively.


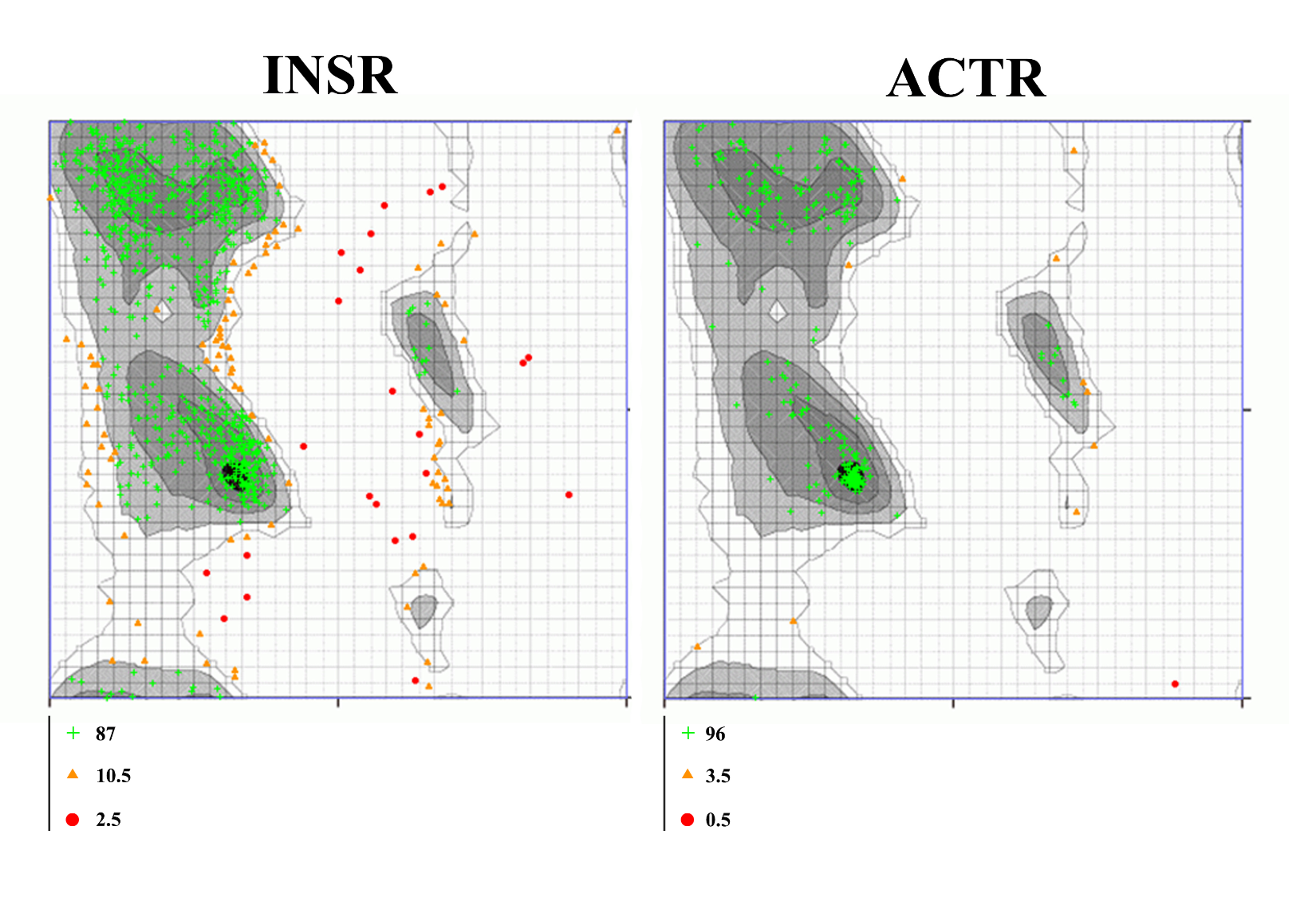


**Supplementary Figure 2.** The three-dimensional structure of INSR depicted by Jmol is based on the reliability of Alphafold predictions. The blue regions indicate the highest reliability (> 93%), and the yellow regions show regions with 60-70% reliability.


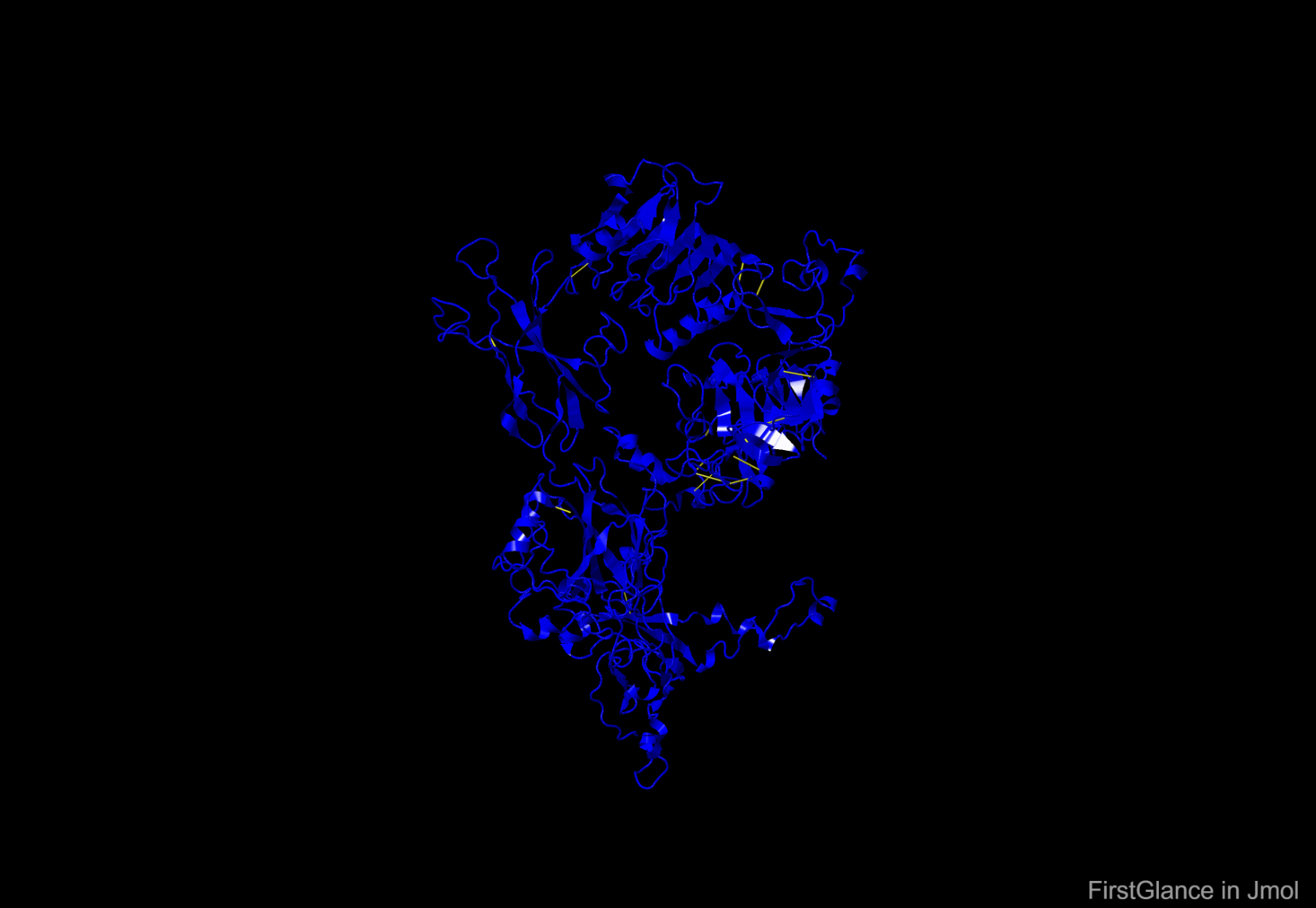


**Supplementary Figure 3**. Composition of the predicted INSR. It has 13.6% helices of all three types (10.1% alpha helices, 3.5% 310 helices, 0% pi helices) and 22.8% and 12.8% beta strands and turns, respectively.


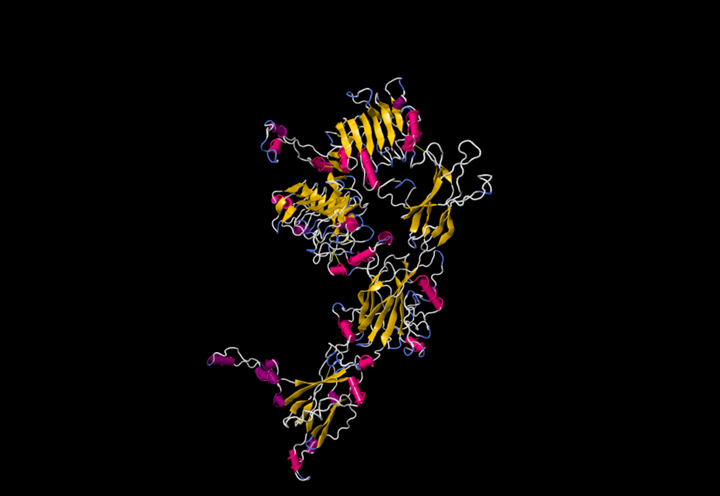

Supplement: Supplementary file 1 [file Data_Sheet_1.docx]
